# Fatal Primary Amebic Meningoencephalitis in Nebraska: Case Report and Environmental Investigation, August 2022

**DOI:** 10.4269/ajtmh.23-0211

**Published:** 2023-07-17

**Authors:** Patrick Maloney, Clayton Mowrer, Lauren Jansen, Tess Karre, Jiri Bedrnicek, Stephen K. Obaro, Peter C. Iwen, Emily McCutchen, Chad Wetzel, Justin Frederick, Muhammad Salman Ashraf, Matthew Donahue

**Affiliations:** ^1^ Nebraska Department of Health and Human Services, Lincoln, Nebraska;; ^2^ University of Nebraska Medical Center, Omaha, Nebraska;; ^3^ Children’s Hospital and Medical Center, Omaha, Nebraska;; ^4^ Centers for Disease Control and Prevention, Division of Workforce Development, Atlanta, Georgia;; ^5^ Nebraska Public Health Laboratory, Omaha, Nebraska;; ^6^ Douglas County Health Department, Omaha, Nebraska

## Abstract

Primary amebic meningoencephalitis (PAM) is a rare and lethal infection caused by *Naegleria fowleri*. We report an epidemiological and environmental investigation relating to a case of PAM in a previously healthy boy age 8 years. An interview of the patient’s family was conducted to determine the likely exposure site and to assess risk factors. Data from the United States Geological Survey site at Waterloo, NE, on the Elkhorn River were used to estimate water temperature and streamflow at the time and site of exposure. Data from the National Weather Service were used to estimate precipitation and ambient air temperature at the time and site of exposure. Despite conventional treatment, the patient died 2 days after hospital admission. The patient participated in recreational water activities in the Elkhorn River in northeastern Nebraska 5 days before symptom onset. In the week before exposure, water and ambient air high temperatures reached annual highs, averaging 32.4°C and 35.8°C, respectively. The day before infection, 2.2 cm of precipitation was reported. Streamflow was low (407 ft^3^/s). Infections in several northern states, including Nebraska, suggest an expanding geographic range of *N. fowleri* transmission, which may lead to increased incidence of PAM in the United States. Similar environmental investigations at suspected exposure sites of future cases will allow data aggregation, enabling investigators to correlate environmental factors with infection risk accurately.

## INTRODUCTION

Primary amebic meningoencephalitis (PAM) is a rare and highly lethal infection caused by *Naegleria fowleri*, a free-living, thermophilic ameba commonly found globally in warm freshwater and soil.^1^ *Naegleria fowleri* is believed to infect humans when water is splashed or inhaled forcibly into the nasal cavity.^2^ Primary amebic meningoencephalitis is associated with recreational water activities such as swimming and diving in warm freshwater sources.^3^

Since 1962, most of the 157 reported cases of PAM in the United States have been localized in the southern states.^4^^–^^6^ Biological and environmental evidence shows that *N. fowleri* tolerates water temperatures as high as 45°C and proliferates at temperatures of 30°C or higher, making the southern region of the United States ideal for *N. fowleri* propagation and transmission.^1^ Additional environmental evidence suggests that precipitation events lead to increased amebic densities in freshwater for the subsequent 1 or 2 days, and transmission occurs most frequently in still or standing water.^3^^,^^7^ Nevertheless, the effects of these environmental factors on human transmission of *N. fowleri* have not been assessed thoroughly. Because of the rarity of PAM, conducting population-based studies to evaluate risk factors for transmission of *N. fowleri* is difficult.

We report the results of epidemiological and environmental investigations of the first reported case of PAM acquired in Nebraska, among the northernmost identified occurrences of disease in the United States.^4^ In doing so, we provide initial information regarding environmental exposures contributing to *N. fowleri* infection and a framework for future epidemiological and environmental investigations.

## CASE REPORT

On the evening of August 15, 2022, a previously healthy white boy (age, 8 years) was admitted to a local hospital with complaints of fever and altered mental status. The patient’s family was interviewed and provided a detailed history of his illness and potential environmental exposures. Symptom onset occurred on August 13, 2022 and included malaise, fatigue, decreased appetite, and mild headache. The following day, the patient experienced worsening headache, onset of fever (38.9°C), and one episode of nonbloody emesis. On August 15, hours before hospital admission, the patient began experiencing altered mental status and unstable ambulation in addition to worsening emesis (three times daily), fever (40.3°C), and headache. The patient also developed a facial rash. This progression of symptoms led the family to seek care in a local emergency department on the evening of August 15 (10:53 pm Central Daylight Time).

According to medical records, the patient’s vital signs on admission to the hospital were as follows: temperature, 38.7°C; heart rate, 120 beats/min; respiratory rate, 44 breaths/min; blood pressure, 125/87 mm Hg; and saturated oxygen, 100%. The patient appeared ill and lethargic. During periods of wakefulness, he experienced disorientation, incoherence, and occasional agitation. Initial laboratory evaluation revealed leukocytosis (white blood cell count, 23.25 cells/μL), and elevated C-reactive protein (1.7 mg/dL) and procalcitonin (11.1 ng/mL). A comprehensive metabolic panel was unremarkable except for an unconjugated bilirubinemia of 3.0 mg/dL. A computed tomographic (CT) scan of the head revealed no acute abnormalities. Blood cultures were obtained on presentation and ultimately were negative. The patient began treatment with empiric ceftriaxone and vancomycin, and was transferred to the pediatric intensive care unit (PICU).

On August 16, a lumbar puncture was performed (12:59 am). The cerebrospinal fluid (CSF) was cloudy and light yellow in appearance, with pleocytosis (6,574 white blood cells/mm^3^ and 91% neutrophilic), glucose < 20 mg/dL, and protein > 600 mg/dL. A gram stain evaluation of CSF did not reveal any organisms. A BioFire FilmArray Meningitis/Encephalitis Panel (bioMérieux, Salt Lake City, UT) was also negative for all pathogens. The patient began exhibiting seizure-like activity followed by disordered breathing, which prompted endotracheal intubation. Worsening tachycardia, persistent fevers, abnormal respiration, and further seizure-like activity followed.

**Figure 1.**
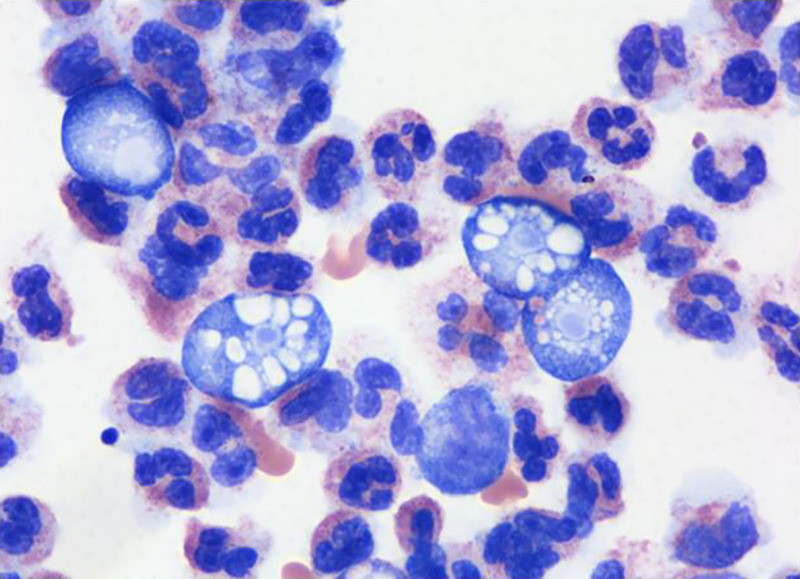
Microscopy of cerebrospinal fluid (100× oil immersion), showing amoeboid trophozoites.

The absence of a definitive etiology, rapid clinical deterioration, and additional microscopic evaluation of the CSF increased suspicion for amebic disease. Empiric therapy was changed to include conventional amphotericin B (one intravenous [IV] dose administered at 1:34 pm, azithromycin (one IV dose administered), fluconazole (one IV dose administered), and rifampin (one IV dose administered). Formal pathological examination with a Wright-Giemsa stain of the CSF revealed the presence of amebic trophozoites (2:30 pm) (Figure 1), and arrangements to obtain miltefosine were made. The CDC was also consulted for diagnostic and treatment guidance (2:25 pm).

Physical examination revealed a new, unequal, fixed pupillary response and absence of cough, gag reflexes, or other voluntary extremity movement. A repeat CT scan of the head without contrast showed new mild diffuse cerebral edema with uncal encroachment on the suprasellar cistern. A subsequent CT scan of the head with contrast showed advancing cerebral edema. The patient was taken to the operating room at 4:37 pm. After failed attempts to place an external ventricular drain, an intracranial pressure monitoring device was placed. After return to the PICU, the patient became hypotensive, requiring inotropic therapy with multiple agents.

Despite these interventions and administration of miltefosine (12:00 am), the patient continued experiencing increasing intracranial pressure and unstable vitals. The patient was extubated terminally and pronounced dead at 3:06 am on August 17, 2022, approximately 28 hours after admission to the hospital and 4 days after symptom onset.

A sample of CSF and images from the pathological examination were sent to the CDC on August 17, 2022. The CDC confirmed that the images were consistent with *N. fowleri* on the same day. The CSF sample was received on August 18, and diagnosis of PAM was confirmed by polymerase chain reaction on August 18, 2022, initiating environmental and epidemiological investigations.

## EPIDEMIOLOGICAL AND ENVIRONMENTAL INVESTIGATION

On August 8, 2022 (5 days before symptom onset), the patient participated in approximately 1 hour (∼3–4 pm) of recreational water activities in the Elkhorn River in Douglas County, NE. These activities included swimming, splashing, and jumping while in the river and on a sandbar located approximately 39.6 m from shore. The family was unsure whether the patient ever submerged his head completely.

Because *N. fowleri* is a thermophilic organism, an association between water temperature and infection might be present.^3^^,^^8^^,^^9^ Obtaining water temperatures before and at the time of exposure might help define more clearly a correlation between temperature and risk for infection, which could guide prevention efforts. To test this association, investigators from the Douglas County Health Department and the Nebraska Department of Health and Human Services conducted an environmental investigation at the suspected site of infection on the Elkhorn River.

At the suspected exposure site, river access is confined to a muddy and reed-choked western bank. A sandbar sits on the eastern side of the river (∼39.6 m from the west bank), stretching to an eastern bank. At the time of investigation, the river measured ∼1.2 m deep at the deepest point. Water clarity was poor at the time of investigation, with suspended sediment impeding visibility.

On August 20, 2022, surface-level and sediment-level temperature readings were taken in the Elkhorn River at the suspected site of infection. Measurements were taken every 6.1 m, beginning at the western bank and terminating at the eastern sandbar (Table 1). Temperatures at surface level and sediment range were 25.8 to 26.2°C and 25.9 to 26.2°C, respectively. Temperature between surface- and sediment-level variation was < 0.1°C.

**Table 1:** Temperature readings at suspected site of infection in the Elkhorn River, Douglas County, NE, 3:00 to 4:00 pm Central Daylight Time, 20 August 2022

Distance from shore, m	Surface temperature, °C	Temperature at sediment, °C
Western shore, 0	26.2	Not measured
6.1	25.8	Not measured
12.2	25.9	25.9
18.3	26.1	26.1
24.4	26.2	26.2
30.5	26.2	26.2
36.6	26.1	26.2
Eastern sandbar, 39.7	26.1	26.2

Given that 12 days interceded exposure and temperature measurements, data obtained from United States Geological Survey (USGS) were used to estimate the water temperature at the time of exposure.^10^ The USGS has a monitoring location that collects data regularly that concern turbidity, water temperature, and more, positioned ∼6.5 km from the exposure site on the Elkhorn River in Waterloo, NE.

**Figure 2.**
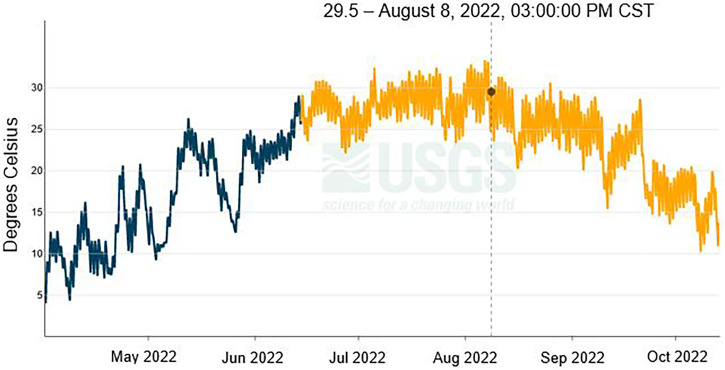
Water temperature readings from the Elkhorn River from April 1, 2022 to October 31, 2022, with estimated time of exposure indicated. United States Geological Survey Waterloo site, 2022.^10^ CST = Central Standard Time.

United States Geological Survey data collected at 3:15 pm on August 20, 2022 (approximate time when temperature readings were taken at the exposure site) recorded a water temperature of 26.2°C (Figure 2). This temperature mirrored the average temperature readings at the exposure site within 0.1°C (26.1°C), indicating that USGS monitoring station temperature recordings approximate the water temperature at the exposure site, despite being approximately 3.1 km away.

The USGS recorded a water temperature of 29.6°C at the approximate time of exposure on August 8, 2022. In the week preceding exposure, the USGS site recorded the highest average high-water temperatures of the year (32.4°C).

**Figure 3.**
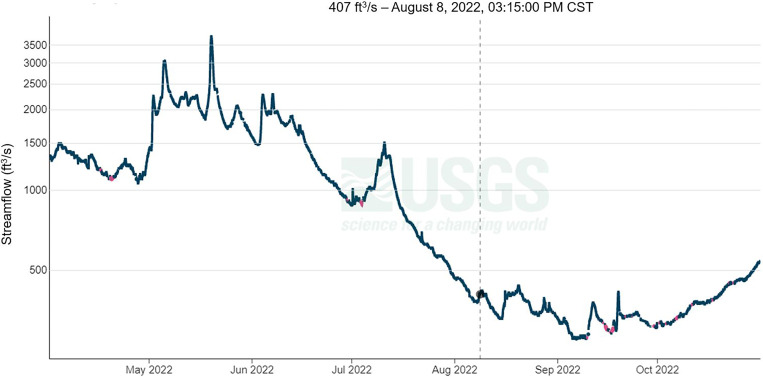
Streamflow readings from the Elkhorn River from April 1, 2022 to October 31, 2022, with estimated time of exposure indicated. United States Geological Survey Waterloo site, 2022.^10^ CST = Central Standard Time.

The USGS also collects data on streamflow, a measure of the rate at which water is carried by rivers and streams. At the estimated time of exposure, the streamflow rate was 407 ft^3^/s (Figure 3). This represented a significant decrease in flowrate from the previous month (1,230 ft^3^/s on July 8, 2022) and began an extended period of low flowrates, which extended through October.

To assess climatological factors, including ambient air temperature and precipitation, publicly available data from the National Weather Services (NWS) were analyzed.^11^ The NWS data were obtained from the Weather Forecast Office in Omaha/Valley, NE, which is located approximately 6.0 km from the exposure site. During the week preceding exposure, the average ambient high air temperature in the area was 35.8°C. According to NWS data, this was the highest average weekly high temperature of 2022. The day before suspected exposure, 2.2 cm of precipitation was reported.

## DISCUSSION

Although *N. fowleri* is pervasive in water and soil in warmer climates, infection is rare.^9^ In a 2021 review of global PAM case reports,^3^ just 182 confirmed cases were identified. (A further 89 probable and 110 expected cases were also reported.) Available data on host factors that may place an individual at increased risk are limited, but do suggest there is an association between male gender and younger age, and infection.^3^^,^^9^ However, because of the disease rarity, ascertaining true causal or contributory factors is difficult. Because of the ubiquity of the ameba in the environment, determining which environmental factors facilitate or inhibit infection is also difficult. The rarity of PAM also precludes the ability to conduct population-based studies analyzing treatment efficacy. The dearth of information regarding PAM is concerning, given that the reported mortality rate ranges from 92% to 99%.^3^^,^^12^ Also, evidence suggests that as global temperatures increase, so will the geographic range of *N. fowleri*, leading to incident cases of PAM in previously unaffected areas.^5^^,^^6^^,^^13^^,^^14^

This case report offers additional insight into the environmental and host factors that could contribute to PAM infection. In the same global review of PAM case reports,^3^ the most common exposure types were swimming or diving (58%), bathing (16%), and water sports (10%). Recreational water activities in this case did include swimming, but the water was shallow, and family members were unable to recall whether the patient’s head was submerged. Although this does not preclude submersion of the patient’s head, it does suggest that submersion was less likely, or at least infrequent. Activities in this case more closely approximated splashing, which made up just 5% of exposures, or 12 cases, globally.^3^

Previous case reports have also identified an association between PAM and prolonged water time in still water sources such as lakes, ponds, and reservoirs.^3^^,^^12^ The family of this patient reported just 1 hour of water exposure, which took place in a river, as opposed to a still water source. River-based exposures have been associated with 8% of global PAM cases.^3^ The limited duration in the water and potential lack of or infrequent submersion of the patient’s head suggests minimal exposure. Combined with the relative rarity of PAM occurrence resulting from river-based exposure, this patient represents a diversion from the typical case profile.

Several environmental factors likely played a role in the transmission of *N. fowleri* in this case. High water (> 32.2°C) and ambient air (> 35°C) temperatures were the highest of the year during the week before infection. In addition, the NWS reported 2.2 cm of precipitation the day before suspected exposure. Last, river streamflow rates were among the lowest of the year (407 ft^3^/s). Because *N. fowleri* thrives in warmer waters, increases in density postprecipitation, and is more highly associated with still or slow-moving water sources, conditions for infection were optimal on August 8, 2022.^1^^,^^3^^,^^7^

## CONCLUSION

This case diverges from typical reports of PAM. Results of epidemiological and environmental investigation suggest that shorter duration of exposure with less-intensive water activities can result in *N. fowleri* infection. In addition, this report indicates that river-based exposures, although rare, do occur. This case was among the northernmost in the United States. The previous identification of PAM cases in northern states and this identification of PAM in Nebraska, suggest that the traditional assumption that PAM is restricted to the southern United States is no longer valid.

Similar environmental and epidemiological investigations will prove essential in identifying factors that promote *N. fowleri* amebic growth and PAM infection. To facilitate earlier diagnosis and early initiation of correct therapy, clinicians should consider PAM as a potential cause of meningitis and encephalitis throughout the United States among those who have had recent exposure to freshwater environments, including rivers. Future case investigations should involve thorough epidemiological and environmental investigations. Through aggregation of data and collaboration among public health practitioners, we can understand more clearly the environmental factors that may be associated with increased risk of *N. fowleri* infection.
