# Fatal Primary Amebic Meningoencephalitis in Nebraska: Case Report and Environmental Investigation, August 2022

**DOI:** 10.4269/ajtmh.23-0211cor

**Published:** 2025-04-02

In the original research paper, “Fatal Primary Amebic Meningoencephalitis in Nebraska: Case Report and Environmental Investigation, August 2022” by Maloney et al., 2023, (https://doi.org/10.4269/ajtmh.23-0211), the authors missed correcting data reported in the Epidemiological and Environmental Investigation section prior to publishing. The positioning of the USGS monitoring location with regard to distance from the exposure site on the Elkhorn River in Waterloo, Nebraska was incorrect. The paper has therefore been corrected. The results and conclusions are unchanged. A summary of the corrected data is shown below:

*“The USGS has a monitoring location that collects data regularly that concern turbidity, water temperature, and more, positioned ∼3.1 km from the exposure site on the Elkhorn River in Waterloo, NE.” (Page 323)*

